# Left ventricular noncompaction and myocardial fibrosis: a case report

**DOI:** 10.1186/1755-7682-3-20

**Published:** 2010-09-15

**Authors:** Jagadeesh K Kalavakunta, Hemasri Tokala, Aparna Gosavi, Vishal Gupta

**Affiliations:** 1 Department of Internal Medicine: Division of Cardiology, Michigan State University/Kalamazoo Center for Medical Studies/Borgess Medical Center, Kalamazoo, MI, USA; 2 Department of Internal Medicine: Division of Cardiology, University of Missouri-Columbia, MO, USA

## Abstract

**Background:**

Left ventricular noncompaction (LVNC) is a rare congenital abnormality. It is currently classified as a genetic cardiomyopathy and results from early arrest of endomyocardial morphogenesis. The pathophysiology of left ventricular dysfunction, which becomes apparent beyond the 4^th ^decade of life, is unclear.

**Case report:**

We report a case of 60-year-old woman who presented with shortness of breath and showed noncompacted endocardium on echocardiography. Cardiac catheterization and viral studies were unremarkable. Histology revealed endomyocardial fibrosis without disarray. She was subsequently diagnosed with LVNC and treated with medications.

**Discussion:**

Cardiologists and other physicians should be aware of LVNC due to its high likelihood of misdiagnosis and associated high complication rates. Early diagnosis, intervention and screening among family members can decrease the morbidity and mortality associated with LVNC.

## Background

Noncompaction of the ventricular myocardium, also called left ventricular noncompaction (LVNC), is a rare congenital abnormality seen in only 0.05% of adults [1]. It is characterized by spongy myocardium and results from arrest of the compaction of the loosely interwoven meshwork of myocardial fibers during endomyocardial morphogenesis between 5-8 weeks of fetal life. With the advent of new diagnostic imaging techniques, more cases of LVNC are being detected. Early diagnosis is crucial due to associated high morbidity and mortality.

## Case Report

A 60-year-old Caucasian woman with a frequent history of asthma, presented to the hospital with several weeks of progressively worsening shortness of breath. She provided a history of intermittent chest pain which, at one time, was relieved with nitroglycerin and morphine, given in the emergency department. As the patient continued having increasing shortness of breath despite adjustments in her asthma medications, she was admitted for further workup. Pertinent positives in her review of systems included decreased appetite, paroxysmal nocturnal dyspnea, orthopnea, lower extremity swelling and intermittent chest pain. The patient denied fever, chills, or cough. Her past medical history was significant for type 2 diabetes, asthma, and osteoarthritis. Medications included theophylline, prednisone, furosemide (Lasix), fluticasone & salmeterol (Advair) and albuterol. She quit smoking 20 years ago and denied alcohol or intravenous drug abuse. Family history was negative for coronary artery disease at an early age. The physical examination was significant for tachycardia, raised jugular venous pressure, lower extremity edema and expiratory wheezes upon chest examination.

**Figure 1:**
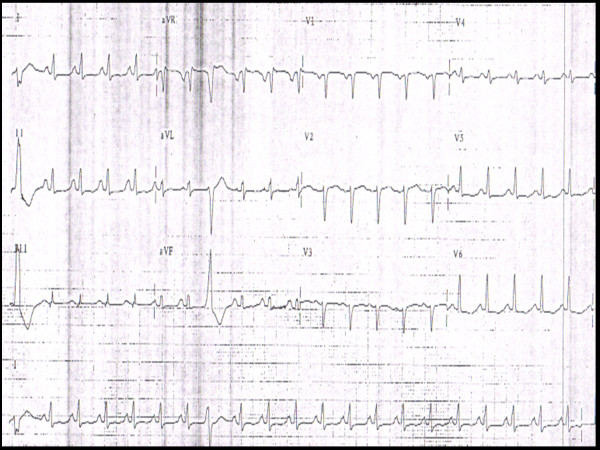
**A 12-lead electrocardiogram showing sinus tachycardia, left atrial enlargement, poor R wave progression and nonspecific ST-T wave changes and T wave inversion in the lateral leads**.

**Figure 2:**
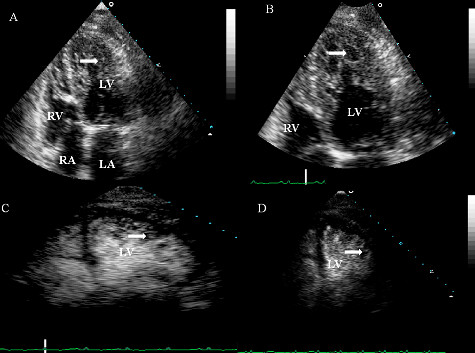
**Transthoracic echocardiogram (A, B, C, D) four chamber view with albumin contrast showing numerous trabeculations (white arrow) in the left ventricular apex, along with deep intertrabecular recesses**. (RA- right atrium, LA- Left atrium, RV-right ventricle, LV- left ventricle).

**Figure 3:**
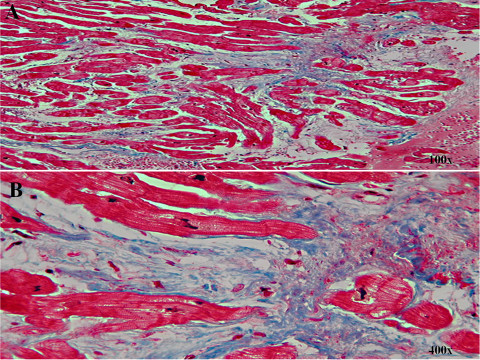
**Endomyocardial biopsy of the left ventricle (hematoxylin and eosin stain) showing the myocardial fibrosis (100×, 400×), along with the cardiac myocytes**.

Laboratory tests revealed elevated brain natriuretic peptide at 1020 pg/ml (normal <100 pg/ml), and negative cardiac enzymes with troponin levels consistently below 0.01 ng/ml (normal 0.00-0.03 ng/ml). Electrocardiogram revealed sinus tachycardia, left atrial enlargement, poor R wave progression and nonspecific ST-T wave changes in all leads specifically T wave inversion in the lateral leads (Figure 1). Chest x-ray showed cardiomegaly with pulmonary vascular congestion. Pulmonary embolism was ruled out by spiral computer tomography (CT) scan. A 2D echocardiogram with albumin echo contrast showed left ventricular (LV) ejection fraction of 25-30% with moderate to severe global hypokinesis of the left ventricle and moderately enlarged left atrium. It also showed a normal sized ventricle with multiple trabeculation in the mid LV cavity and apex suggesting either an apical form of hypertrophic cardiomyopathy or LVNC (Figure 2). She underwent cardiac catheterization which revealed normal coronary arteries. In view of the normal coronaries and severe global hypokinesis, further workup was done to rule out other causes of cardiomyopathy. Viral cultures were negative for enteric cytopathic human orphan [ECHO] and coxsackie viruses. To further elucidate the cause of the cardiomyopathy, LV endomyocardial biopsy was performed. Histology showed myocardial fibrosis suggestive of cardiomyopathy, possibly secondary to LVNC (Figure 3).

## Discussion

Left ventricular noncompaction is a rare cause of cardiomyopathy, and patients present with systolic dysfunction commonly of the left, and sometimes of the right ventricle. The incidence of LVNC in clinical practice is low because it is an under-recognized phenomenon and most cases are diagnosed as idiopathic cardiomyopathy. Age of onset and degree of clinical symptoms depend on the extent of the noncompacted cardiac segments [2].

The exact pathophysiology of the ventricular dysfunction is not known. Jenni, et al, suggested subendocardial hypoperfusion and microcirculatory dysfunction in development of ventricular dysfunction and arrhythmogenesis [3]. Echocardiography is the reference standard for diagnosing LVNC, although it can be well defined by CT scan, positron emission tomography and magnetic resonance imaging [4-8]. Echocardiogram shows a two-layered hypokinetic myocardium with thin, compacted epicardium, thick, noncompacted endocardium, and a noncompaction (NC) to compaction (C) ratio >2, and blood flowing directly from the ventricular cavity in to the deep intertrabecular recess [2,9]. In this case the NC/C was >2 with multiple trabeculations and it could be classified into the prominent trabeculation morphological group of LVNC [10].

LVNC is genetically heterogeneous, with predominance of autosomal dominant inheritance, and is currently classified under cardiomyopathies as a genetic disease [11]. LVNC leading to myocardial fibrosis has not been well defined in the literature [12]. The etiology of the fibrosis is unclear, but it explains the LV dysfunction associated with LVNC. Recent studies have reported a significant prevalence of mitochondrial myopathy and genetic mutations in patients with LVNC. Myopathies with cardiac involvement have also been implicated in LVNC, including Barth syndrome, Emery-Dreifuss muscular dystrophy, and myotubular myopathy [13,14]. Genetic mutations, especially the alpha-dystrobrevin gene, Cypher/ZASP and gene G4.5 of the Xq28 chromosome region and loss of the cardiac-specific gene CSX have been well described in the literature as causes of LVNC [15-17]. These genetic mutations and myopathies may eventually lead to fibrosis of the myocardium causing severe LV dysfunction. This process may take several years, thus explaining why heart failure in patients with LVNC usually occurs beyond the 4^th ^decade of life. LVNC is misdiagnosed or missed in a majority of the cases due to its varied presentation and is not associated with any specific histological finding [18]. In a small case series, Burke, et al, found that LVNC was associated with endocardial fibroelastosis and anastomosing or polypoid endocardial trabeculations [19]. In our case, biopsy showed myocardial fibrosis without any disarray. Absence of disarray in the histology excluded hypertrophic cardiomyopathy in this case.

Clinical manifestation of LVNC ranges from absence of symptoms to disabling congestive heart failure, arrhythmias, such as atrial fibrillation, ventricular tachyarrhythmias, and sudden cardiac death, and thromboembolic events [20]. LVNC association with neuromuscular disorders is commonly seen with Barth syndrome and mitochondrial disorders [21]. With its varied presentation and genetic association, patients diagnosed with LVNC warrant genetic counseling, DNA diagnostics, and cardiological family screening to make an early diagnosis and prevent complications [22].

Treatment is symptomatic, including consideration of heart transplantation. Medical treatment depends mainly upon the clinical presentation. Cardiac resynchronization therapy (CRT) or placement of an implantable cardioverter-defibrillators (ICD) should be considered when the LV ejection fraction ≤35% and prior to heart transplantation. In one study, the largest cohort of LVNC patients showed the effectiveness of ICD for secondary or primary prevention of sudden cardiac death, and improvement of New York Heart Association (NYHA) class in patients with LV ejection fraction ≤35% or LVNC with CRT [23]. There are no concrete guidelines for anticoagulation therapy as a primary prevention of thromboembolism among LVNC patients. Many authors believe that it should be considered among LVNC patients with atrial fibrillation, severe LV dysfunction, or a history of thromboembolism. In our case, implantation of an ICD, warfarin anticoagulation was offered to the patient but she refused.

Prognosis, in general, is poor and improves significantly with heart transplantation. A larger case series of 34 patients with long-term follow-up of 44 months, showed mortality of 35%, and with sudden cardiac death accounting for 50% of fatalities [2]. In the same study, 24% of patients had thromboembolic events and 41% had ventricular tachyarrhythmias. A recent similar study showed lower mortality and fewer complications [24]. This variation is secondary to the inclusion of milder/preclinical cases in the later study, along with advancement in the echocardiographic technology that facilitate earlier diagnosis. Heart transplantation is considered only when medical therapy fails to control the progression of heart failure. Our patient responded well to treatment with angiotensin-converting enzyme inhibitors and beta blockers.

## Conclusion

Cardiologists and other physicians should be aware of LVNC due to its high likelihood of misdiagnosis and consequent high complication rates. Early diagnosis and intervention, and screening among family members can decrease the morbidity and mortality associated with LVNC.

## Consent

Written informed consent was obtained from the patient for publication of this case report. A copy of the written consent is available for review by the Editor-in-Chief of this journal.

## Competing interests

The authors declare that they have no competing interests.

## Authors' contributions

All the four authors contributed to the conception, design, analysis and preparation of the manuscript. All authors read and approved the final manuscript.
